# Children's Diarrhœa

**Published:** 1887-07-23

**Authors:** 


### Children's Diarrhœa.

This is the season for infantile diarrhœa, and the disease is much more serious than many people suppose. By some a slight attack of looseness of the bowels in a child is considered a good thing; by many such an occurrence is regarded as of little moment, and often allowed to continue for three or four days before medical aid is sought. Even then it is generally the extremely debilitated condition of the little patient which causes anxiety, rather than the actual diarrhœa. In hot weather this disease is always serious, and especially so in young children. At the very outset of the symptoms medical aid should be sought. A delicate child may be so reduced in a day or two, or even in a few hours by a severe attack, that recovery may be almost impossible. In towns especially, where the tenacity of life is much less decided than in the country, prompt remedial measures should be taken. We do not give any suggestions for remedies, because it often happens that parental tinkering causes just sufficient loss of time to turn an otherwise simple and easily cured case into a dangerous or even fatal one. We do advise, however, that judicious care be exercised with regard to milk, fruit, vegetables, and other articles of diet which decompose readily in the extreme heat. Great watchfulness should be observed, too, in protecting children from the dangerous vertical rays of the mid-day sun.

